# Is ypTNM staging a comparable predictor as pTNM staging for survival in non-metastatic rectal cancer after preoperative chemoradiation therapy?

**DOI:** 10.32604/or.2024.052098

**Published:** 2024-10-16

**Authors:** JEN-PIN CHUANG, HSIANG-LIN TSAI, WEI-CHIH SU, PO-JUNG CHEN, CHING-WEN HUANG, TSUNG-KUN CHANG, YEN-CHENG CHEN, CHING-CHUN LI, YUNG-SUNG YEH, TZU-CHIEH YIN, JAW-YUAN WANG

**Affiliations:** 1 Ministry of Health and Welfare, Chiayi Hospital, Chiayi, 60096, Taiwan; 2 Department of Surgery, Faculty of Medicine, College of Medicine, National Cheng Kung University, Tainan, 70101, Taiwan; 3 Department of Surgery, National Cheng Kung University Hospital, Tainan, 70101, Taiwan; 4 Division of Colorectal Surgery, Department of Surgery, Kaohsiung Medical University Hospital, Kaohsiung Medical University, Kaohsiung, 80708, Taiwan; 5 Department of Surgery, Faculty of Medicine, College of Medicine, Kaohsiung Medical University, Kaohsiung, 80708, Taiwan; 6 Graduate Institute of Clinical Medicine, College of Medicine, Kaohsiung Medical University, Kaohsiung, 80708, Taiwan; 7 Department of Surgery, Faculty of Post-Baccalaureate Medicine, College of Medicine, Kaohsiung Medical University, Kaohsiung, 80708, Taiwan; 8 Division of Colorectal Surgery, Department of Surgery, Kaohsiung Municipal Hsiaokang Hospital, Kaohsiung, 80708, Taiwan; 9 Division of Trauma and Surgical Critical Care, Department of Surgery, Kaohsiung Medical University Hospital, Kaohsiung Medical University, Kaohsiung, 80708, Taiwan; 10 Department of Emergency Medicine, Faculty of Post-Baccalaureate Medicine, College of Medicine, Kaohsiung Medical University, Kaohsiung, 80708, Taiwan; 11 Department of Surgery, Kaohsiung Municipal Tatung Hospital, Kaohsiung, 80145, Taiwan; 12 Graduate Institute of Medicine, College of Medicine, Kaohsiung Medical University, Kaohsiung, 80708, Taiwan; 13 Center for Cancer Research, Kaohsiung Medical University, Kaohsiung, 80708, Taiwan

**Keywords:** pTNM, ypTNM, Rectal cancer, Inverse probability treatment weighting, Survival

## Abstract

**Background:** The pTNM staging system is widely recognized as the most effective prognostic indicator for cancer. The latest update of this staging system introduced a new pathological staging system (ypTNM) for patients receiving neoadjuvant chemoradiotherapy (NACRT). However, whether the prognostic value of the ypTNM staging system for rectal cancer is similar to that of the pTNM staging system remains unclear. This study was conducted to compare the ypTNM and pTNM staging systems in terms of their prognostic value for patients with nonmetastatic rectal cancer undergoing proctectomy. **Material and Methods:** This study was conducted at a large teaching hospital. Between January 2014 and December 2022, 542 patients with rectal cancer were analyzed (median follow-up period, 60 months; range, 6–105 months). Of them, 258 and 284 were included in the pTNM and ypTNM groups, respectively. Inverse probability of treatment weighting (IPTW) was performed to account for the effects of confounders. Cox proportional-hazards regression was performed for the between-group comparison of overall survival (OS). **Results:** The crude model revealed that OS was similar between the two groups (*p* = 0.607). After performing IPTW, we found that patients with the same ypTNM- and pTNM-classified stages had similar overall survival (hazard ratio = 1.15; 95% CI = 0.76–1.73; *p* = 0.5074). **Conclusions:** For patients with rectal cancer who have received preoperative NACRT, the prognostic value of ypTNM staging appears to be similar to that of pTNM staging, mostly because of the downstaging effect of neoadjuvant chemoradiotherapy.

## Introduction

Colorectal cancer (CRC) is the second leading cause of cancer-related mortalities in both men and women worldwide. According to the Global Cancer Observatory report published by the World Health Organization, more than 1.9 million new cases of CRC were reported globally in 2020. Among these cases, approximately 732,210 were new diagnoses of rectal cancer [1]. Currently, preoperative neoadjuvant chemoradiotherapy (NACRT) followed by radical resection is the standard therapy for patients with stage II/III carcinoma of the rectum [2–6].

According to the Surveillance, Epidemiology, and End Results Program of the National Cancer Institute, the overall 5-year survival rates for rectal cancer at the localized, regional, and distant metastasis stages were 90%, 73%, and 17%, respectively, between 2011 and 2017 [7]. The Tumor, Node, and Metastasis (TNM) staging system remains the most effective tool for predicting the prognosis of rectal cancer. In addition to the pTNM staging system, the ypTNM staging system, which represents the pathologic tumor response after preoperative NACRT, was introduced in the latest version (eighth) of the pTNM staging guidelines issued by the American Joint Commission on Cancer (AJCC) in October 2016 [8]. In clinical practice, pathological staging is regarded as the most effective prognostic indicator for patients with rectal cancer [9,10]; however, this conclusion was drawn before the introduction of ypTNM. Furthermore, because of the downstaging effect of NACRT, the pTNM and ypTNM stages of patients not receiving preoperative NACRT are expected to be considerably different, although they are classified under the same stage.

Two clinical questions must be addressed. The first question is whether the new ypTNM staging system is prognostically as effective as the pTNM staging system for rectal cancer treated with NACRT. The second question is whether patients receiving preoperative NACRT and those not receiving it should be managed with the same treatment regimen if they have the same pathological stage. Few studies have explored the aforementioned questions; therefore, we conducted this retrospective study to compare the ypTNM and pTNM staging systems in terms of their prognostic value for patients with nonmetastatic rectal cancer undergoing proctectomy.

## Materials and Methods

### Patients

Between 2014 and 2022, 641 patients with rectal cancer were identified as potential participants for the present study. Patients were eligible if they had received a preoperative pathological diagnosis of rectal adenocarcinoma (through tissue biopsy), had complete clinical and postoperative pathological data available, and had undergone radical proctectomy. Patients were excluded if they had received a diagnosis of gastrointestinal stromal tumor, lymphoma, neuroendocrine tumor, carcinoid tumor, soft tissue tumor, or other nonrectal adenocarcinomas before treatment. In addition, patients with stage IV rectal malignancies, those who died within 1 month after surgery, those who had received chemoradiotherapy for other neoplasms within 6 months before rectal cancer surgery, and those who received a diagnosis of rectal cancer at the age of ≤19 years were excluded from this study.

Before treatment, all participants underwent a series of pretreatment assessments, including physical examination, medical history review, colonoscopy, tumor biopsy, chest radiography, abdominal computed tomography, pelvic magnetic resonance imaging, and routine laboratory analysis. In the clinical records of the participants, their pTNM tumor stages were classified in accordance with the seventh edition of the AJCC Cancer Staging Manual and Handbook [11]. Patients who had received preoperative NACRT were classified using the ypTNM staging system in accordance with the Eighth Edition of the AJCC Cancer Staging Manual [12]. The present study was approved by the Institutional Review Board of Kaohsiung Medical University Hospital (approval number: KMUHIRB-E(I)-20210041).

### Preoperative therapy

Patients with T3, T4, or N+ rectal cancer received preoperative NACRT in accordance with our previous protocol [13]. Radiotherapy was administered to the entire pelvis at a dose of 45 Gy in 25 fractions, followed by a 5.4-Gy boost to the primary tumor in three fractions. The concurrent chemotherapy (CCRT) regimen comprised the biweekly administration of mFOLFOX6 in combination with radiotherapy. Patients in the study underwent the administration of a total of 12 cycles of the FOLFOX regimen, with a split between neoadjuvant and adjuvant chemotherapy phases. Prior to surgery, patients underwent six to seven cycles of FOLFOX, followed by the remaining cycles post-surgery. Each cycle of FOLFOX included oxaliplatin (85 mg/m^2^) on the first day, folinic acid (400 mg/m^2^), and a 46-h infusion of 5-FU (2800 mg/m^2^) every two weeks. Surgical intervention took place approximately 10 to 12 weeks after the completion of radiotherapy. The participants who had been diagnosed as having cT2 rectal cancer within 4 cm from the anal verge also received preoperative NACRT following the same regimen. Subsequently, the participants underwent standard total mesorectal excision within 10–12 weeks after the completion of radiotherapy [13].

### Surgery

For tumors situated in the upper and mid rectum, low-anterior resection (LAR) with the double-stapled technique was employed as the surgical approach. In instances of low rectal cancers (tumor located in less than 4 cm from anal verge), intersphincteric resection was undertaken, followed by transanal extraction and resection of the specimen (utilizing natural orifice specimen extraction). Hand-sewn coloanal anastomosis was conducted, with the creation of a loop colostomy. These surgical interventions were executed through open, laparoscopic, or robotic surgery techniques depends on shared-decision making.

### Adjuvant chemotherapy

According to the guidelines established within our institution, stage II with unfavorable features or stage III locally advanced rectal cancer patients are recommended to undergo adjuvant therapy following NACRT and subsequent radical resection. Specifically, those with postoperative pathological findings indicating a positive primary tumor (ypT+) or positive regional lymph nodes (ypN+) are advised to complete a total of 12 cycles of the FOLFOX regimen, inclusive of cycles administered prior to surgical intervention. Conversely, patients achieving a pathological complete response postoperatively are prescribed fluoropyrimidine-based chemotherapy for a duration of up to 3 months following surgery.

### Outcome and confounders

The primary outcome of interest in the present study was overall survival (OS), which was defined as the period between the initial diagnosis of rectal cancer and either the date of all-cause mortality or the latest follow-up. In addition, the participants’ demographic, clinical, and pathological data were extracted from a database and analyzed as potential predictors or confounders of OS; these data included the participants’ age, sex, body mass index, Eastern Cooperative Oncology Group (ECOG) score, cancer history, resection margin, pathology T and N stages, differentiation grade, and comorbidities (e.g., diabetes mellitus, hypertension, coronary artery disease, cerebrovascular accident, chronic kidney disease (CKD), tuberculosis, chronic obstructive pulmonary disease, and asthma). Furthermore, health hazard behaviors such as betel quid chewing, cigarette smoking, and alcohol drinking were also considered in the analysis, as presented in Table 1.

**Table 1:** Clinical and pathological characteristics of patients with rectal cancer undergoing proctectomy with or without preoperative neoadjuvant chemoradiotherapy (N = 542)

Characteristics	Total N = 542	pTNM n = 258	ypTNM n = 284	*p* value*
Age (μ ± SD)	61.5 ± 11.8	61.2 ± 12.4	61.8 ± 11.2	0.573
Female	226 (41.7%)	115 (44.6%)	111(39.1%)	0.196
Follow-up (month)	60.4 ± 26.2	60.4 ± 27.2	60.5 ± 25.3	0.9
History of cancer (yes)	30 (5.5%)	15 (5.8%)	15 (5.3%)	0.787
BMI (median ± SD, kg/m^2^)	26.8 ± 13.2	26.5 ± 9.1	27 ± 16	0.67
ECOG				0.114
0	204 (37.6%)	106 (41.1%)	98 (34.5%)	
1	338 (62.4%)	152 (58.9%)	186 (65.5%)	
Resection margin free	541 (99.8%)	257 (99.6%)	284 (100%)	0.294
Differentiation grade				0.171
Well	40 (7.4%)	23 (8.9%)	17 (6%)	
Moderately	477 (88%)	220 (85.3%)	257 (90.5%)	
Poor	25 (4.6%)	15 (5.8%)	10 (3.5%)	
T stage				<0.001*
Tis	108 (19.9%)	16 (6.2%)	92 (32.4%)	
T1	98 (18.1%)	75 (29.1%)	23 (8.1%)	
T2	111 (20.5%)	52 (20.2%)	59 (20.8%)	
T3	211 (38.9%)	107 (41.5%)	104 (36.6%)	
T4a	10 (1.8%)	8 (3.1%)	2 (0.7%)	
T4b	4 (0.7%)	0 (0.0%)	4 (1.4%)	
N stage				<0.001*
N0	398 (73.4%)	174 (67.4%)	224 (78.9%)	
N1	13 (2.4%)	9 (3.5%)	4 (1.4%)	
N1a	46 (8.5%)	23 (8.9%)	23 (8.1%)	
N1b	34 (6.3%)	23 (8.9%)	11 (3.9%)	
N1c	21 (3.9%)	5 (1.9%)	16 (5.6%)	
N2a	18 (3.3%)	14 (5.4%)	4 (1.4%)	
N2b	12 (2.2%)	10 (3.9%)	2 (0.7%)	
TNM stage				<0.001*
0	107 (19.7%)	16 (5.7%)	91 (26.9%)	
I	170 (31.4%)	102 (36.6%)	68 (20.1%)	
IIA	115 (21.2%)	53 (19.0%)	62 (18.3%)	
IIB	3 (0.6%)	2 (0.7%)	1 (0.3%)	
IIC	2 (0.4%)	0 (0.0%)	2 (0.6%)	
IIIA	37 (6.8%)	23 (8.2%)	14 (4.1%)	
IIIB	93 (17.2%)	51 (18.3%)	42 (12.4%)	
IIIC	15 (2.8%)	11 (3.9%)	4 (1.2%)	
DM	130 (24%)	59 (22.9%)	71 (25%)	0.562
HTN	210 (38.7%)	103 (39.9%)	107 (37.7%)	0.592
CAD	21 (3.9%)	8 (3.1%)	9 (3.2%)	0.964
CVA	15 (2.8%)	8 (3.1%)	7 (2.5%)	0.652
CKD	21 (3.9%)	15 (5.8%)	6 (2.1%)	0.026*
TB	2 (0.4%)	0 (0%)	2 (0.7%)	0.177
COPD	4 (0.7%)	2 (0.8%)	2 (0.7%)	0.923
Asthma	9 (1.7%)	7 (2.7%)	2 (0.7%)	0.068
Cigarette smoking	66 (12.2%)	21 (8.1%)	45 (15.8%)	0.006*
Betel quid chewing	12 (2.2%)	6 (2.3%)	6 (2.1%)	0.866
Alcohol consumption	40 (7.4%)	12 (4.7%)	28 (9.9%)	0.021*

Note: *p < 0.05. Abbreviations: ECOG, Eastern Cooperative Oncology Group; DM, diabetes mellitus; HTN, hypertension; CAD, coronary artery disease; CVA, cerebrovascular accident; CKD, chronic kidney disease; TB, tuberculosis; and COPD, chronic obstructive pulmonary disease.

### Statistical analysis

Descriptive statistics are presented in terms of frequencies for categorical variables and means ± standard deviations for continuous variables. Chi-square and Fisher’s exact tests were performed to compare categorical data; for continuous variables, analyses were performed using Student’s *t* and Mann–Whitney U tests for normally and nonnormally distributed data, respectively. The Kaplan–Meier method was used to determine the crude OS rate, and a log-rank test was performed to compare time-to-event distributions. Statistical significance was set at *p* < 0.05, and all *p* values were two-tailed. The analyses were performed using SPSS for Windows (version 25.0; SPSS Inc., Chicago, IL, USA).

To enhance the credibility of overall cohort findings, propensity scores were computed using confounding factors as adjustment variables. A logistic regression model with preoperative NACRT as the dependent variable was developed, and all variables presented in Table 2 were used as explanatory variables. To evaluate causal treatment effects, unbiased estimates of average treatment effects were obtained through 1:1 inverse probability of treatment weighting (IPTW) [14]. To trim high weights, asymmetric truncation was performed using 99th percentile as the threshold for downward trimming [15]. After IPTW, a survival analysis was performed using the weight of a Cox proportional-hazards regression model to compare the pTNM and ypTNM groups in terms of survival distribution. Hazard ratios (HRs) with 95% confidence intervals (CIs) were calculated to estimate the risk of mortality. These analyses were performed using SAS (version 9.6; SAS Institute, Cary, NC, USA).

**Table 2:** Clinical and pathological characteristics of the pTNM and ypTNM groups after applying inverse probability of treatment weighting (N = 1065)

Characteristics	pTNM (N = 531)	ypTNM (N = 534)	*p* value
Age (μ ± SD)	61.3 ± 12.2	61.6 ± 11.4	0.733
Female	44.6%	45.3%	0.822
Previous cancer history	4.1%	4.9%	0.564
BMI (median ± SD, kg/m^2^)	26 ± 8.8	26.5 ± 15.9	0.554
ECOG			0.924
0	37.9%	37.6%	
1	62.1%	62.4%	
Resection margin free	99.8%	100.0%	0.316
Differentiation grade			0.821
Well	8.1%	7.1%	
Moderately	86.1%	86.7%	
Poor	5.8%	6.2%	
T stage			0.313
Tis	18.1%	20.3%	
T1	18.5%	18.6%	
T2	21.5%	20.7%	
T3	40.1%	38.5%	
T4a	1.9%	1.1%	
T4b	0%	0.8%	
N stage			0.922
N0	74.3%	75.3%	
N1	2.5%	2.1%	
N1a	7.7%	8.4%	
N1b	6.2%	5.6%	
N1c	4.0%	4.6%	
N2a	3.6%	3.9%	
N2b	2.3%	1.3%	
DM	22.2%	22.1%	0.974
HTN	35.6%	37.6%	0.488
CAD	2.6%	2.4%	0.834
CVA	2.8%	2.6%	0.843
CKD	4.3%	3.9%	0.744
TB	0.0%	0.4%	0.158
COPD	0.6%	0.6%	0.994
Asthma	1.7%	1.5%	0.798
Cigarette smoking	12.6%	13.5%	0.675
Betel quid chewing	2.5%	2.1%	0.669
Alcohol consumption	8.7%	8.3%	0.811

Abbreviations: ECOG, Eastern Cooperative Oncology Group; DM, diabetes mellitus; HTN, hypertension; CAD, coronary artery disease; CVA, cerebrovascular accident; CKD, chronic kidney disease; TB, tuberculosis; and COPD, chronic obstructive pulmonary disease.

## Results

### Patient characteristics

**Figure 1:**
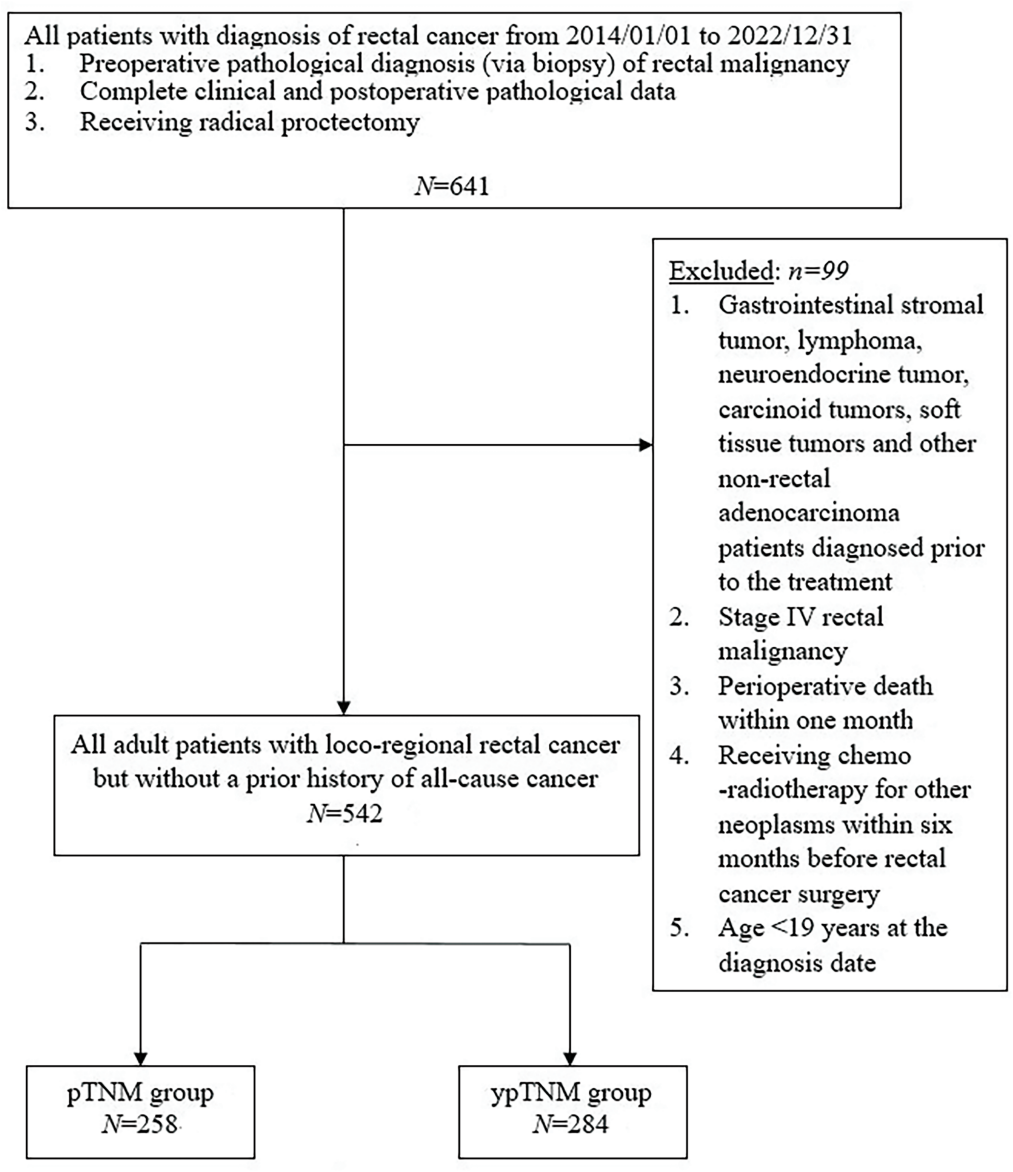
Protocol for the recruitment of study participants.

A total of 641 eligible patients were initially included in the present study. Among them, 99 were subsequently excluded, resulting in a final cohort of 542 patients. Of them, 258 and 284 patients were assigned to the pTNM and ypTNM groups, respectively (Fig. 1). The clinicopathological characteristics of the unmatched cohort are presented in Table 1. The mean age of the participants was 61.5 ± 11.8 years, and 226 of them (41.7%) were women. Among the participants, 31.4%, 19.7%, 22.1%, and 26.8% had stage I, stage 0, stage II, and stage III cancer, respectively. All participants had an ECOG performance status of 0 or 1, and only 5.5% had a history of nonrectal malignancy. Of the participants, 99.8% underwent R0 resection. Rectal adenocarcinoma of most patients had a moderate differentiation grade (88%) with a pT or ypT stage of T3 (38.9%) and an ypN or pN stage of N0 (73.4%). The median duration of follow-up for all patients, the ypTNM group, and the pTNM group was 58.5 (range = 6–105), 58.5 (range = 7–105), and 58 (range = 6–105) months, respectively. Regarding comorbidities, the pTNM group had a significantly higher proportion of participants with CKD than did the ypTNM group (5.8% *vs*. 2.1%, respectively; *p* = 0.026). The analysis of negative health promotion behaviors revealed that the ypTNM group had higher proportions of cigarette smokers and alcohol drinkers than did the pTNM group (cigarette smokers, 15.8% *vs*. 8.1%, respectively [*p* = 0.006]; alcohol drinkers, 9.9% *vs*. 4.7%, respectively [*p* = 0.021]).

### Survival analysis

**Figure 2:**
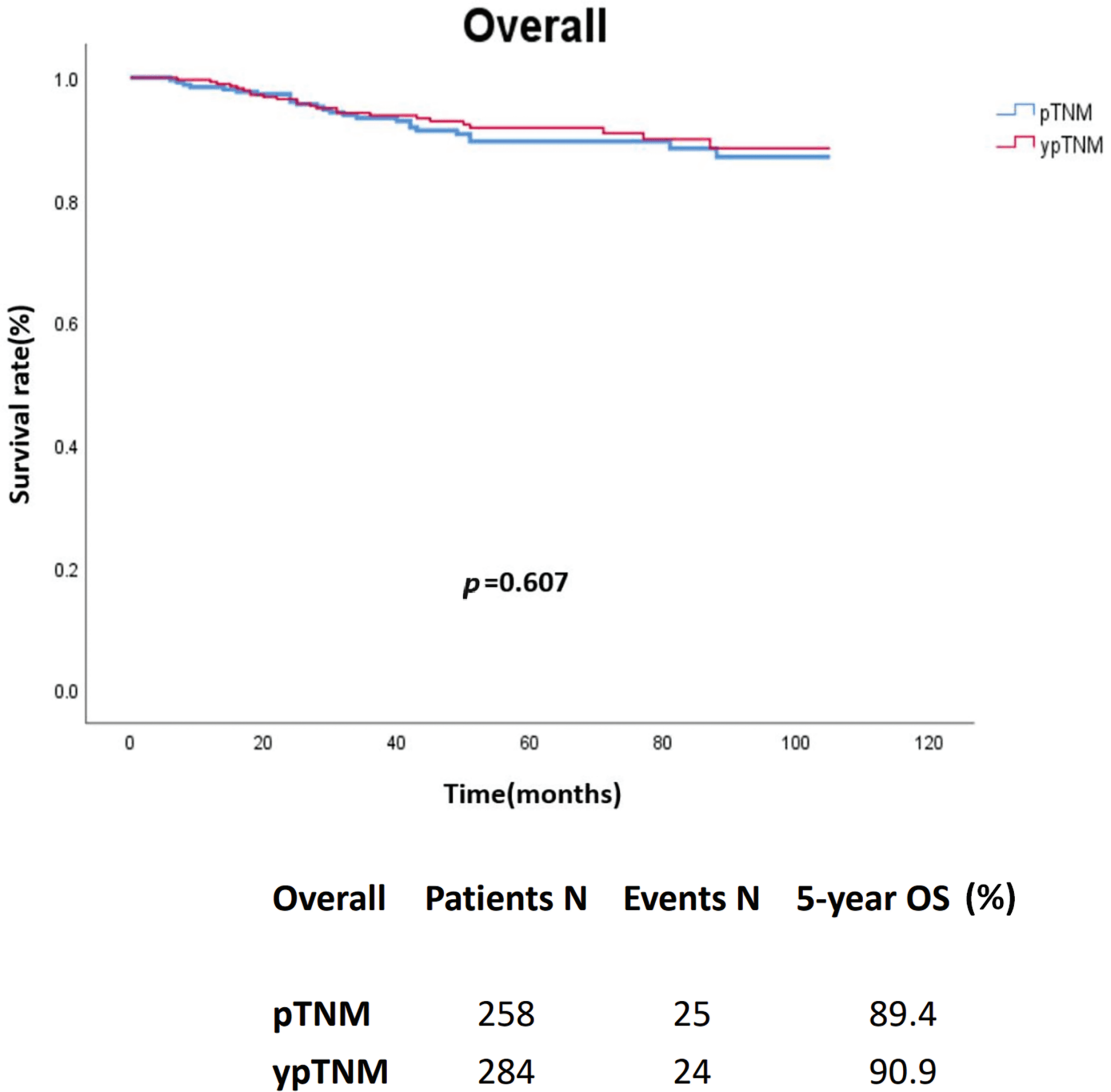
Crude overall survival of the unmatched cohort of patients with rectal cancer; pTNM (N = 258) *vs*. ypTNM (N = 284).

**Figure 3:**
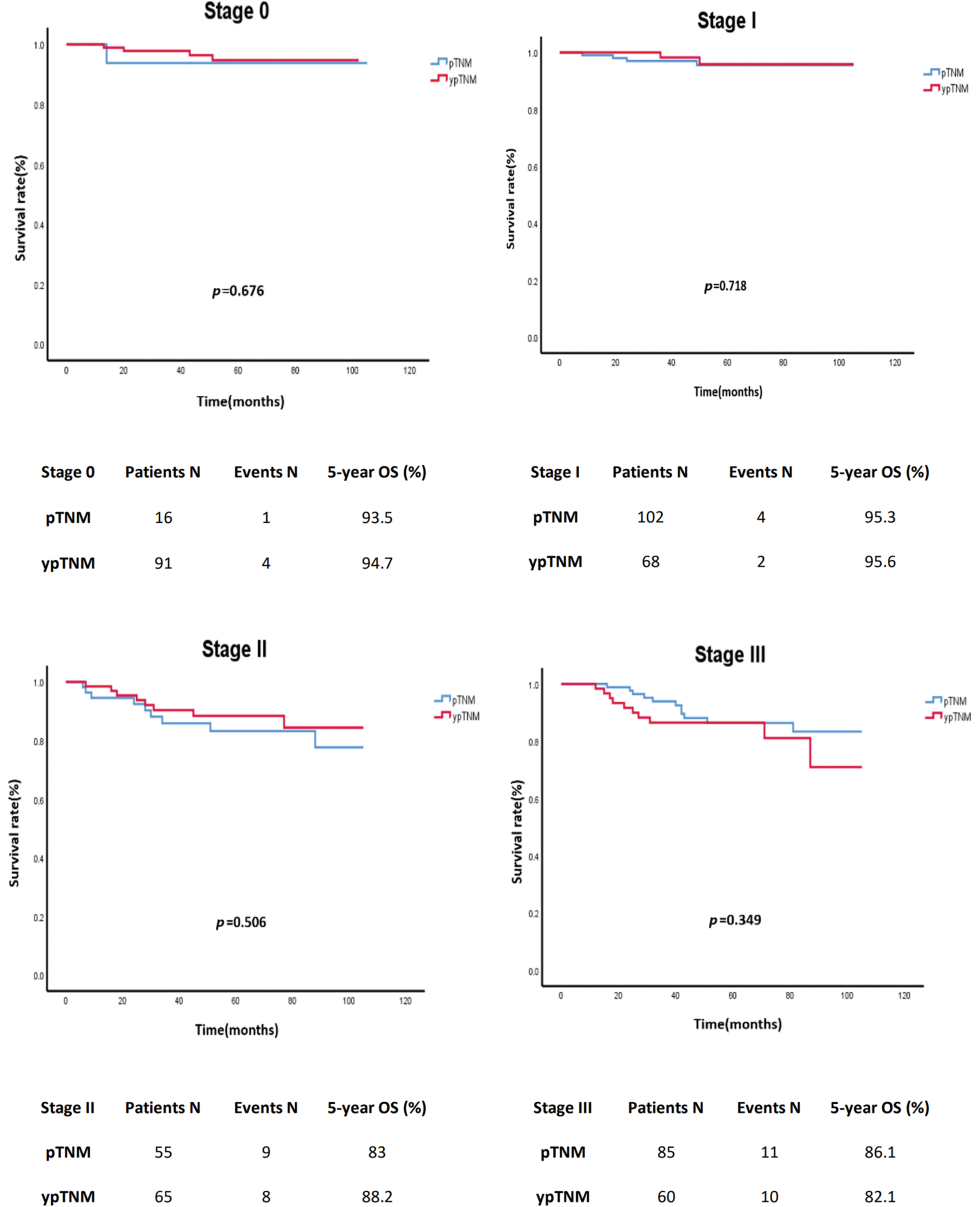
Crude overall survival of the unmatched cohort of patients with rectal cancer stratified by TNM stage; pTNM (N = 258) *vs*. ypTNM (N = 284).

In an unmatched analysis, no significant difference was observed in survival rate between the pTNM and ypTNM groups (*p* = 0.607). The 5-year crude OS rates for all patients, the pTNM group, and the ypTNM group were 90.2%, 89.4%, and 90.9%, respectively (Fig. 2). A subgroup analysis (by TNM stage) performed using Kaplan–Meier data revealed that the pTNM and ypTNM groups had similar survival outcomes (Fig. 3).

### Characteristics of the post-IPTW cohort

IPTW was performed to adjust for the effects of potential confounders and ensure similar distributions for the T and N stages between the two groups. This step ensured that the measured covariates were approximately random and that no confounder was associated with the treatment of interest. The post-IPTW sample comprised 1065 patients (531 and 534 in the pTNM and ypTNM groups, respectively). Table 2 lists the postadjustment differences in covariates between the pTNM and ypTNM groups. All previously observed covariate imbalances between the two groups became nonsignificant after IPTW adjustment, and the distribution of factors associated with the hazard of all-cause mortality became balanced. The clinicopathological characteristics of the matched cohort were similar to those of the unmatched cohort, with most participants in both groups having T3 in T stage and N0 in N stage.

### Post-IPTW survival analysis

The analysis of the post-IPTW cohort using a Cox proportional-hazards model revealed an HR value of 1.149 for the comparison between the pTNM and ypTNM groups (95% CI = 0.762–1.733; *p* = 0.507). A subgroup analysis indicated that the similarity between the ypTNM and pTNM groups in terms of OS remained consistent for individual TNM stages. For patients with stage 0 rectal cancer and those with stage I rectal cancer, the HR values were 0.972 (95% CI = 0.2–4.79; *p* = 0.972) and 2.27 (95% CI = 0.80–6.45; *p* = 0.126), respectively. Similarly, for patients with stage II rectal cancer and those with stage III rectal cancer, the HR values were 0.728 (95% CI = 0.35–1.51; *p* = 0.39) and 1.249 (95% CI = 0.63–2.48; *p* = 0.52), respectively. Overall, our findings revealed no significant difference; after IPTW, the pTNM and ypTNM groups were found to have similar OS (Table 3).

**Table 3:** Survival analysis (Cox proportional-hazards models) of all-cause mortality after applying inverse probability of treatment weighting for each TNM stage

Stage	Post IPTW adjustment sample size		HR	95% CI	*p* value
	pTNM	ypTNM			
Stage 0	85	104	0.972	0.197–4.787	0.972
Stage I	168	162	2.266	0.796–6.453	0.126
Stage II	114	109	0.728	0.351–1.510	0.393
Stage III	132	124	1.249	0.630–2.476	0.525
Overall	531	534	1.149	0.762–1.733	0.507

## Discussion

Over the past two decades, preoperative CCRT has become the standard NACRT for locally advanced rectal cancer [3,5]. The latest edition of the AJCC Cancer Staging Manual introduces the ypTNM classification system, which considers the severity of cancer after therapy. Although several studies have focused on the importance of neoadjuvant therapy in predicting cancer prognosis [9,16,17], the pTNM stage remains the predominant predictor of OS. The present study was conducted to investigate whether patients with the same ypTNM- and pTNM-classified stages of rectal cancer exhibit similar survival outcomes. To the best of our knowledge, this is the first study to address this clinical question in the context of rectal cancer.

### Patients with ypTNM- and pTNM-classified stage 0 or I rectal cancer

Our findings revealed that OS was similar between patients with ypTNM- and pTNM-classified stage 0 or I rectal cancer. Typically, patients with pTNM-classified stage 0 or I rectal cancer did not undergo preoperative NACRT, whereas most patients with ypTNM-classified stage 0 or I rectal cancer were downstaged from stages II or III after a favorable response to NACRT. If ypTNM-classified patients with stage 0 or I rectal cancer had not received NACRT, their pathological stage would have been higher than 0 and 1 and they would have had more unfavorable outcomes than those of pTNM-classified patients with stage 0 or I rectal cancer. Nevertheless, studies have indicated that posttreatment pathologic TNM stage is correlated with post-NACRT disease-free survival and tumor recurrence rate in locally advanced rectal cancer [18,19]. Furthermore, a complete pathologic response to neoadjuvant treatment confers oncologic benefits in terms of both overall recurrence and disease-free survival [9,16,17,20]. We previously demonstrated that a partial tumor response to preoperative chemoradiotherapy against rectal cancer is associated with a 50% increase in disease-free survival and regarded as a favorable prognostic factor [3]. These findings may explain why the downstaged patients in our study achieved the same oncological outcome as those with pTNM-classified early-stage cancer.

### Patients with ypTNM-classified stage II or III rectal cancer

The participants with ypTNM-classified stage II rectal cancer comprised two distinct subsets. The first subset comprised patients who were downstaged from stage III through NACRT, whereas the second subset comprised those who did not respond adequately to NACRT. The combination of these two subsets yielded a nonsignificant HR. The participants with ypTNM-classified stage III rectal cancer did not respond favorably to preoperative NACRT and, therefore, remained in stage III. Such patients were previously assumed to have a worse prognosis than that noted in patients with pTNM-classified stage III rectal cancer because patients with ypTNM-classified stage III rectal cancer were regarded as insensitive to chemotherapy. However, the difference in prognosis between the pTNM and ypTNM groups was nonsignificant in our study, which further confirmed the beneficial effects of preoperative NACRT, even for patients who were not downstaged. We previously reported that for locally advanced rectal cancer, post-NACRT carcinoembryonic antigen is the predominant predictor of complete pathologic response, followed by the interval between preoperative NACRT and surgery, chemotherapy regimens, clinical nodal stage, and clinical tumor stage [21]. Another study suggested that locally advanced rectal cancer with mismatch-repair deficiency is exceptionally sensitive to single-agent programmed cell death-1 blockade, which is a key mediator of immune suppression (within the tumor microenvironment) in NACRT [22].

### Analysis with IPTW

IPTW is a statistical method used to construct comparable groups for investigating the effects of a treatment or exposure. In contrast to methods that involve matching treated and untreated individuals since a select set of confounders, IPTW considers the entire cohort and can address numerous confounders. By assigning weights to all individuals in a cohort based on the likelihood of their exposure to a treatment, researchers can minimize or eliminate the effects of confounders during statistical analysis or regression modeling. We performed IPTW to adjust for the potential confounders of treatment outcomes.

### Strengths and limitations

The present study has several advantages, including its single-center design, which ensured the consistent treatment and testing of all participants. Consequently, the risk of data variability was minimized, which enabled us to draw conclusive findings. Additionally, a single team of health-care professionals administered preoperative CCRT to all patients and performed their surgery, which ensured strict quality control over the study design as well as data collection and analysis.

Our study has some limitations. Specifically, it was conducted retrospectively at a single center with a small sample size. The reported number of events for each stage of the study seems inadequate, particularly in stages two and three, where the event counts are approximately ten. The scarcity of events is due to the insufficient sample size resulting from further categorization by different pathological stages. This constrained sample size may impact the study’s statistical power and the extent to which its findings can be generalized. In addition, the validity of the ypTNM and pTNM systems, as assessed by the differential survivals observed at each stage, could not be adequately demonstrated in our study primarily due to an insufficient sample size. The study lacked a sufficient number of cases to further address uncontrolled factors among the different stages. Apart from the stage of the tumor, new evidence confirms that people with colorectal cancer (CRC) who have stable microsatellites (MSS) and mutations in BRAF or KRAS genes tend to have worse survival rates [23]. However, complete pathologic molecular biomarker data were not available for all participants. Hence, we could not perform a multivariate analysis for each pathology stage and adjust for factors such as MSI, EGFR, K-RAS, and ERCC1. These factors have the potential to affect the outcomes of NACRT and thus should be considered in future studies.

## Conclusions

In this study, the overall and subgroup analyses revealed no significant difference in survival outcomes between patients with ypTNM-classified and pTNM-classified stages of rectal cancer. This trend was consistent across all TNM stages. The similar survival outcomes among patients with comparable pathological stages pertains to the effectiveness of downstaging following NACRT. In the absence of NACRT, the prognosis for patients with a more advanced stage is expected to be poorer. Our findings suggest that preoperative NACRT is a reliable neoadjuvant therapy for patients with nonmetastatic rectal cancer. To improve the reliability and generalizability of our findings, future studies should ensure collaborations among multiple institutions to increase the sample size and statistical power.

## Data Availability

The datasets used and/or analyzed during the current study are available from the corresponding author on reasonable request.

## References

[1] Sung H, Ferlay J, Siegel RL, Laversanne M, Soerjomataram I, Jemal A, et al. Global Cancer Statistics 2020: gLOBOCAN estimates of incidence and mortality worldwide for 36 cancers in 185 countries. CA A Cancer J Clin. 2021;71(3):209–49. doi:10.3322/caac.v71.3. 33538338

[2] Camma C, Giunta M, Fiorica F, Pagliaro L, Craxi A, Cottone M. Preoperative radiotherapy for resectable rectal cancer: a meta-analysis. JAMA. 2000;284(8):1008–15. doi:10.1001/jama.284.8.1008; 10944647

[3] Lee YC, Hsieh CC, Chuang JP. Prognostic significance of partial tumor regression after preoperative chemoradiotherapy for rectal cancer: a meta-analysis. Dis Colon Rectum. 2013;56(9):1093–101. doi:10.1097/DCR.0b013e318298e36b; 23929020

[4] Smith CA, Kachnic LA. Evolving treatment paradigm in the treatment of locally advanced rectal cancer. J Nat Compr Cancer Netw JNCCN. 2018;16(7):909–15. doi:10.6004/jnccn.2018.7032; 30006431

[5] Huang MY, Lee HH, Tsai HL, Huang CW, Yeh YS, Ma CJ, et al. Comparison of efficacy and safety of preoperative chemoradiotherapy in locally advanced upper and middle/lower rectal cancer. Radiat Oncol. 2018;13(1):53. doi:10.1186/s13014-018-0987-0; 29587797 PMC5870751

[6] Lin H, Wang L, Zhong X, Zhang X, Shao L, Wu J. Meta-analysis of neoadjuvant chemotherapy versus neoadjuvant chemoradiotherapy for locally advanced rectal cancer. World J Surg Oncol. 2021;19(1):141. doi:10.1186/s12957-021-02251-0; 33952287 PMC8101236

[7] Siegel RL, Miller KD, Fuchs HE, Jemal A. Cancer statistics, 2022. CA A Cancer J Clin. 2022;72(1):7–33. doi:10.3322/caac.v72.1. 35020204

[8] Weiser MR. AJCC 8th edition: colorectal cancer. Ann Surg Oncol. 2018;25(6):1454–5. doi:10.1245/s10434-018-6462-1; 29616422

[9] Quah HM, Chou JF, Gonen M, Shia J, Schrag D, Saltz LB, et al. Pathologic stage is most prognostic of disease-free survival in locally advanced rectal cancer patients after preoperative chemoradiation. Cancer. 2008;113(1):57–64. doi:10.1002/cncr.v113:1. 18442099

[10] Brierley JD, Greene FL, Sobin LH, Wittekind C. The “y” symbol: an important classification tool for neoadjuvant cancer treatment. Cancer. 2006;106(11):2526–7. doi:10.1002/cncr.v106:11. 16596656

[11] Edge SB, Byrd DR, Carducci MA, Compton CC, Fritz A, Greene F. AJCC cancer staging manual. 7th edition. Available from: https://www.facs.org/media/j30havyf/ajcc_7thed_cancer_staging_manual.pdf. [Accessed 2010].

[12] Amin MB, Edge S, Byrd D. AJCC cancer staging manual. 8th ed. NYC: Springer; 2017.

[13] Huang CM, Huang MY, Tsai HL, Huang CW, Ma CJ, Yeh YS, et al. An observational study of extending FOLFOX chemotherapy, lengthening the interval between radiotherapy and surgery, and enhancing pathological complete response rates in rectal cancer patients following preoperative chemoradiotherapy. Ther Adv Gastroenterol. 2016;9(5):702–12. doi:10.1177/1756283X16656690; 27582883 PMC4984334

[14] Austin PC, Stuart EA. Moving towards best practice when using inverse probability of treatment weighting (IPTW) using the propensity score to estimate causal treatment effects in observational studies. Stat Med. 2015;34(28):3661–79. doi:10.1002/sim.v34.28. 26238958 PMC4626409

[15] Lee BK, Lessler J, Stuart EA. Weight trimming and propensity score weighting. PLoS One. 2011;6(3):e18174. doi:10.1371/journal.pone.0018174; 21483818 PMC3069059

[16] Jang TY, Yu CS, Yoon YS, Lim SB, Hong SM, Kim TW, et al. Oncologic outcome after preoperative chemoradiotherapy in patients with pathologic T0 (ypT0) rectal cancer. Dis Colon Rectum. 2012;55(10):1024–31. doi:10.1097/DCR.0b013e3182644334; 22965400

[17] Chan AK, Wong A, Jenken D, Heine J, Buie D, Johnson D. Posttreatment TNM staging is a prognostic indicator of survival and recurrence in tethered or fixed rectal carcinoma after preoperative chemotherapy and radiotherapy. Int J Radiat Oncol Biol Phys. 2005;61(3):665–77. doi:10.1016/j.ijrobp.2004.06.206; 15708244

[18] Huang MY, Huang CW, Wang JY. Surgical treatment following neoadjuvant chemoradiotherapy in locally advanced rectal cancer. Kaohsiung J Med Sci. 2020;36(3):152–9. doi:10.1002/kjm2.v36.3. 31814296 PMC11896401

[19] Huang CM, Huang MY, Tsai HL, Huang CW, Su WC, Chang TK, et al. Pretreatment neutrophil-to-lymphocyte ratio associated with tumor recurrence and survival in patients achieving a pathological complete response following neoadjuvant chemoradiotherapy for rectal cancer. Cancers. 2021;13(18):4589. doi:10.3390/cancers13184589; 34572816 PMC8470001

[20] Kuo LJ, Liu MC, Jian JJ, Horng CF, Cheng TI, Chen CM, et al. Is final TNM staging a predictor for survival in locally advanced rectal cancer after preoperative chemoradiation therapy? Ann Surg Oncol. 2007;14(10):2766–72. doi:10.1245/s10434-007-9471-z; 17551794

[21] Huang CM, Huang MY, Huang CW, Tsai HL, Su WC, Chang WC, et al. Machine learning for predicting pathological complete response in patients with locally advanced rectal cancer after neoadjuvant chemoradiotherapy. Sci Rep. 2020;10(1):12555. doi:10.1038/s41598-020-69345-9; 32724164 PMC7387337

[22] Yeh YS, Tsai HL, Chen PJ, Chen YC, Su WC, Chang TK, et al. Identifying and clinically validating biomarkers for immunotherapy in colorectal cancer. Exp Rev Mol Diagn. 2023;23(3):231–41. doi:10.1080/14737159.2023.2188195; 36908268

[23] Alwers E, Blaker H, Walter V, Jansen L, Kloor M, Arnold A, et al. External validation of molecular subtype classifications of colorectal cancer based on microsatellite instability, CIMP, BRAF and KRAS. BMC Cancer. 2019;19(1):681. doi:10.1186/s12885-019-5842-7; 31296182 PMC6624952

